# 

**Published:** 1881-02

**Authors:** 


### CONTENTS.

### COMMUNICATIONS.

Therapeutical Requisites Necessary to the Salvation of every Dental Practitioner .... 47
Chronic Chloral Poisoning .... 51
Some Medical Quacks .... 55
The Determination of Normal Vision .... 59

### SELECTIONS.

What has become of the Metric System? .... 62
The Treatment of Insomnia .... 63
Case of Hemorrhagic Diathesis .... 64
The Metric System in Machine Shops .... 65
Ether and Chloroform .... 66
The Bogus Diploma Business in Pennsylvania .... 67
Headache in School Children; Difference in the Estimate of Longevity .... 68
Guilding .... 69
The Use of Chlorophyl in Vegetable Growth; Dangers of "Bonwill's Method" of Anæsthesia .... 70
Electric Light good for the Eyes .... 71
Quillaia Toothwash; To Render Ivory Flexible; Perfumed Carbolic Acid .... 72
Longevity of the Friends .... 73
Effects of an Overdose of Belladonna; Correspondence .... 74
The Goodyear Vulcanite Co. and the Rubber Patents .... 78

### EDITORIAL.

Is it a College? .... 80
Soft Foil .... 82
International Medical Congress .... 84
About Coin .... 85
The Sad End of a Promising Life; A New Broach .... 86
Ohio State Dental Society .... 87
Alumni Meeting; Iowa State Dental Society; Married .... 88

